# Interstitial Telomeric Repeats Are Rare in Turtles

**DOI:** 10.3390/genes11060657

**Published:** 2020-06-16

**Authors:** Lorenzo Clemente, Sofia Mazzoleni, Eleonora Pensabene Bellavia, Barbora Augstenová, Markus Auer, Peter Praschag, Tomáš Protiva, Petr Velenský, Philipp Wagner, Uwe Fritz, Lukáš Kratochvíl, Michail Rovatsos

**Affiliations:** 1 Department of Ecology, Faculty of Science, Charles University, 12844 Prague, Czech Republic; lorenzo.clemente@natur.cuni.cz (L.C.); sofia.mazzoleni@natur.cuni.cz (S.M.); pensabee@natur.cuni.cz (E.P.B.); barbora.augstenova@natur.cuni.cz (B.A.); kratoch1@natur.cuni.cz (L.K.); 2 Museum of Zoology, Senckenberg Dresden, 01109 Dresden, Germany; Markus.Auer@senckenberg.de (M.A.); uwe.fritz@senckenberg.de (U.F.); 3 Turtle Island, 8041 Graz, Austria; ppraschag@turtle-island.at; 4 landsnail.org, 14200 Prague, Czech Republic; info@landsnails.org; 5 Prague Zoological Garden, 17100 Prague, Czech Republic; velensky@zoopraha.cz; 6 Allwetterzoo Münster, D48161 Münster, Germany; wagner@allwetterzoo.de

**Keywords:** evolution, FISH, in situ hybridization, ITRs, interstitial telomeric repeats, ITSs, interstitial telomeric sequences, karyotype, telomeres, turtles

## Abstract

Telomeres are nucleoprotein complexes protecting chromosome ends in most eukaryotic organisms. In addition to chromosome ends, telomeric-like motifs can be accumulated in centromeric, pericentromeric and intermediate (i.e., between centromeres and telomeres) positions as so-called interstitial telomeric repeats (ITRs). We mapped the distribution of (TTAGGG)_n_ repeats in the karyotypes of 30 species from nine families of turtles using fluorescence in situ hybridization. All examined species showed the expected terminal topology of telomeric motifs at the edges of chromosomes. We detected ITRs in only five species from three families. Combining our and literature data, we inferred seven independent origins of ITRs among turtles. ITRs occurred in turtles in centromeric positions, often in several chromosomal pairs, in a given species. Their distribution does not correspond directly to interchromosomal rearrangements. Our findings support that centromeres and non-recombining parts of sex chromosomes are very dynamic genomic regions, even in turtles, a group generally thought to be slowly evolving. However, in contrast to squamate reptiles (lizards and snakes), where ITRs were found in more than half of the examined species, and birds, the presence of ITRs is generally rare in turtles, which agrees with the expected low rates of chromosomal rearrangements and rather slow karyotype evolution in this group.

## 1. Introduction

Telomeres are regions of repetitive DNA motifs and associated proteins localized at the edges of chromosomes. They play a crucial role in maintaining the chromosome structure as a single unit, preventing fusions of free “sticky” chromosome ends, or degeneration and loss of genetic information during the replication events [1]. In vertebrates, telomeric regions consist of long tandem repeats of the TTAGGG motif, which was first characterized in humans [2] and seems to be extremely conserved across all studied vertebrates [3]. Telomeres are formed and preserved by telomerase, a reverse transcriptase able to add new TTAGGG repeats based on an RNA template and to compensate for the normal shortening of telomeres after each replication event [4,5,6,7]. However, the presence and, therefore, activity of telomerase varies across cell types and developmental stages [8]. Notably, the shortening of the telomeres is associated with cell aging and cell death [9,10,11,12]. Interestingly, (TTAGGG)_n_ motifs can be found also in non-terminal positions of the chromosomes [3,13,14] in the form of interstitial telomeric repeats (ITRs) also referred to as interstitial telomeric sequences (ITSs). Some authors classified ITRs into two main groups: the short ITRs (s-ITRs) and the heterochromatic ITRs (Het-ITRs) [15]. Other authors report a more detailed classification including s-ITRs, subtelomeric ITRs, fusion ITRs and pericentromeric ITRs [14,15,16,17,18]. s-ITRs are characterized by a low copy number of (TTAGGG)_n_ tandem repeats, located in interstitial positions of the chromosomes. The bloom of genome sequencing projects revealed that s-ITRs are common in vertebrate genomes [19,20,21,22,23,24,25,26]. They are often not detectable by molecular cytogenetic methods, for example by fluorescence in situ hybridization (FISH), due to the low number of repeats [15]. In contrast, Het-ITRs consist of an extensive accumulation of (TTAGGG)_n_ tandem repeats mainly located in heterochromatic regions, particularly in centromeric and pericentromeric areas, but occasionally also interspersed within chromosome arms. Het-ITRs are less common than s-ITRs in vertebrate genomes and are often detectable by molecular cytogenetic methods [15,27,28,29,30]. In our study, we classify ITRs into three categories: centromeric, pericentromeric and intermediate ITRs, according to their position following our previous classification for squamate reptiles [28]. 

The origin and function of ITRs still remain unclear. According to the most prevalent hypothesis, ITRs are remnants of former terminal telomeric sequences that have been repositioned to interstitial position as a consequence of chromosomal fusions [3], fissions and inversions. Especially Het-ITRs are linked to chromosomal rearrangements [15], and their presence in centromeric and pericentromeric regions are often taken as a marker of former chromosomal fusions [22,31,32]. Alternatively, ITRs can emerge in hotspots for DNA breakage and recombination [14,17], produced by the cell repair machinery after unequal crossing over and double-strand breaks [33,34,35,36]. This last hypothesis explains the origin of s-ITRs, and it is supported by the observation that telomerase is able to insert telomeric repeats into double strand break sites during chromosome healing [37,38]. Notably, long regions of ITRs can also emerge via amplification of pre-existing short (TTAGGG)_n_ repeats present in the genomes as latent telomeres [3]. 

Cytogenetic studies have detected ITRs in numerous species across vertebrate lineages, including mammals, fishes, birds, non-avian reptiles and amphibians [3,27,28,29,30,39,40,41,42,43,44,45,46]. In non-avian reptiles, distribution of telomeric sequences have been extensively studied in squamates, i.e., lizards and snakes, where ITRs were detected in approximately 100 species, despite the generally conserved chromosome morphology in this group [28,45,47,48,49,50,51,52,53,54,55,56,57,58,59,60,61,62,63,64]. It was proposed that intrachromosomal rearrangements might have a crucial role in the formation of ITRs in squamate reptiles [28,63]. A telomere-only pattern was instead reported in *Crocodylus siamensis* [65], and as far as we know, no other representative of Crocodylia has been studied. In birds, ITRs are relatively common and mostly found in centromeric and pericentromeric positions [39,44,66,67]. Notably, in some cases, the telomeric motif TTAGGG is part of a larger satellite DNA sequence, and its presence and amplification in the genome is not directly connected to chromosomal rearrangements [68,69]. In turtles, the distribution of telomeric motifs have been studied up to our knowledge in 27 out of the 353 extant species [70,71], but ITRs have been previously reported in only six species [72,73,74,75,76,77,78,79,80,81,82], reviewed further in this study, but more systematic survey is needed before drawing solid conclusions on the frequency of ITRs in turtles.

Turtles have diploid chromosome numbers ranging from 2n = 26 to 2n = 68 [76,83]; however, diploid numbers and chromosome morphology seem to be conserved and rather stable among species within a family [76,84,85]. Phylogenetic reconstruction suggested that the ancestral turtle karyotype was composed of 2n = 52 chromosomes [76]. The variability in chromosome numbers across turtles is driven mainly by fusions involving microchromosomes leading to reduction of diploid chromosome numbers (e.g., in the ancestor of the families Pelomedusidae and Podocnemididae), and by chromosome fissions leading to increase of diploid chromosome numbers (e.g., in the ancestor of the families Carettochelyidae and Trionychidae) [76]. 

In this study, we explored the distribution of telomeric repeats from representative species across the turtle phylogeny by analyzing 30 species from nine families using fluorescence in situ hybridization with a probe specific for the telomeric repeats. We combine our results with previously published data, and present an overview of the distribution of ITRs across turtles and compare their frequency to other reptile lineages.

## 2. Materials and Methods

### 2.1. Sampling

We studied the distribution of telomeric (TTAGGG)_n_ repeats in the karyotypes of 30 turtle species representing nine families (Table 1). Blood samples were collected from captive-bred or legally imported turtles from the Münster Zoo (Germany), Plzeň Zoo, Prague Zoo (both Czech Republic), the Museum of Zoology, Senckenberg Dresden (Germany), Turtle Island (Austria) and private keepers. Experiments with animals were performed by accredited researchers (LK: CZ02535, MR: CZ03540). When needed, turtles were temporarily kept in the animal facility of the Faculty of Science, Charles University (accreditation No. 37428/2019-MZE-18134). The species identity of studied turtles was verified in morphologically challenging taxa using mtDNA sequences that were compared to the sequences published in the revisionary works by Fritz et al. [86] for the genus *Cyclemys*, Petzold et al. [87] for *Pelomedusa*, and Ihlow et al. [88] for *Malayemys*. The Galápagos giant tortoises used in the present study from the Prague Zoo are of known provenance and species identity. All experimental procedures were carried out under the supervision and with the approval of the Ethics Committee of the Faculty of Science, Charles University, approved by the Committee for Animal Welfare of the Ministry of Agriculture of the Czech Republic (permit No. MSMT-34426/2019-7).

### 2.2. Chromosome Preparation and Staining

Chromosome suspensions were prepared from whole blood cell cultures. Briefly, the cultivation medium consisted of 90 mL of D-MEM medium (Sigma-Aldrich, St. Louis, MO, USA), enriched with 10 mL of fetal bovine serum (GIBCO, Carlsbad, CA, USA), 3 mL of phytohemagglutinin M (GIBCO, Carlsbad, CA, USA), 1 mL of penicillin/streptomycin solution (10,000 units/mL; GIBCO, Carlsbad, CA, USA), 1 mL L-glutamine solution (200 mM; Sigma-Aldrich, St. Louis, MO, USA) and 1 mL lipopolysaccharide solution (10 mg/mL; Sigma-Aldrich, St. Louis, MO, USA). Then, 100–300 μL of blood samples were added to the 5 mL of cultivation medium and incubated for one week at 30 °C, without CO_2_ supplementation. After the incubation period, the cultures were first treated with 35 μL of colcemid (10 μg/mL; Roche, Basel, Switzerland) and incubated for 3 h and 30 min at 30 °C. The cells were treated with a pre-warmed 0.075 M KCl hypotonic solution for 30 min at 37 °C, washed and fixed four times with cold 3:1 methanol/acetic acid solution. The chromosome suspensions were later stored at −20 °C for further use.

### 2.3. Giemsa Staining and Karyotype Reconstruction

Metaphases from species with undescribed karyotypes were stained with 8% Giemsa solution. Selected metaphases were captured using a Zeiss Axio Imager Z2 (Zeiss, Oberkochen, Germany), equipped with a Metafer-MSearch automatic scanning platform (MetaSystems, Altlussheim, Germany) and CoolCube 1 b/w digital camera (MetaSystems, Altlussheim, Germany). Karyograms were prepared using the Ikaros karyotyping platform (MetaSystems, Altlussheim, Germany). At least 10 metaphases per individual were studied.

### 2.4. Fluorescence In Situ Hybridization with Probes for Telomeric Repeats

The (TTAGGG)_n_ probe for telomeric sequences was prepared and labelled with dUTP-biotin by PCR, using the (TTAGGG)_5_ and (CCCTAA)_5_ primers, without DNA template following the protocol of Ijdo et al. [89] and Rovatsos et al. [27]. The probe was diluted in a hybridization buffer (50% formamide in 2 × SSC, pH 7) and stored in the freezer for further use.

The slides with chromosome spreads were aged either overnight at 37 °C or for 1 h at 60 °C. The FISH experiment was conducted in two days. During the first day, the chromosome spreads were treated with RNase (100 μg/mL) for 1 h at 37 °C, 0.01% pepsin for 10 min at 37 °C, post-fixed in 1% formaldehyde solution for 10 min and dehydrated in a 70–85–95% ethanol series. Once dried, the slides were denatured in 70% formamide for 4 min at 75 °C and dehydrated once more with the ethanol series. Meanwhile, the probe was denatured for 6 min at 73 °C and kept in ice for at least 10 min. To each slide, 10 μL of probe was added, covered with a coverslide and incubated overnight at 37 °C. During the second day, the slides were washed 3 times with 50% formamide/2 × SSC solution for 5 min at 37 °C, two times with 2 × SSC for 5 min and once with 4 × SSC/0.05% Tween 20 (Sigma-Aldrich, St. Louis, MO, USA) for 5 min. The slides were incubated in 4 × SSC/5% blocking reagent (Roche) for 45 min at 37 °C and then in 4 × SSC/5% blocking reagent containing avidin-FITC (Vector laboratories, Burlingame, CA, USA) for 30 min at 37 °C. The fluorescence signal was twice amplified by the fluorescein–avidin D/biotinylated anti-avidin system (Vector Laboratories, Burlingame, CA, USA). After this treatment, the slides were dehydrated in ethanol series, air-dried and stained with Fluoroshield with DAPI (Sigma-Aldrich, St. Louis, MO, USA).

For each specimen, at least 20 images were obtained using a Provis AX70 (Olympus, Tokyo, Japan) fluorescence microscope equipped with a DP30BW digital camera (Olympus, Tokyo, Japan). The pictures were obtained in BW and later superimposed in color with DP Manager imaging software (Olympus, Tokyo, Japan).

### 2.5. Distribution of ITRs across the Turtle Phylogeny

**Figure 1:**
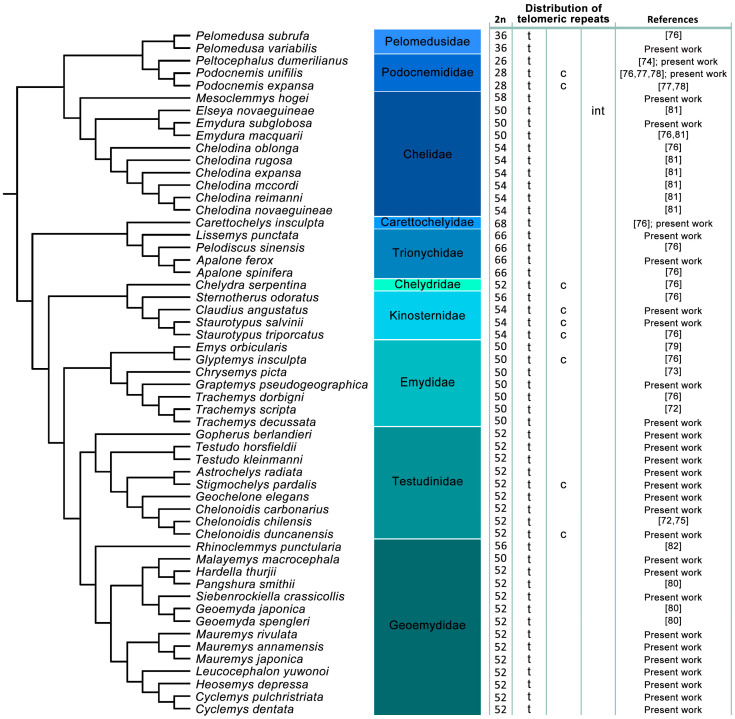
Distribution of the presence of telomeric repeats across turtles. Telomeric repeats were detected in terminal topology (t) in all studied species of turtles, and additionally, interstitial telomeric repeats (ITRs) were identified in centromeric (c) (9 species) and intermediate (int) (1 species) positions. ITRs identification follows our previous classification for squamate reptiles by Rovatsos et al. [28]. Phylogeny follows Pereira et al. [90] and Kehlmaier et al. [91].

Data for the topology on the karyotype of telomeric sequences from the species studied here was supplemented by previously published records in order to reconstruct the phylogenetic history of the presence/absence of ITRs in turtles (Figure 1). The phylogenetic trees by Pereira et al. [90] and Kehlmaier et al. [91] were used for this phylogenetic reconstruction in the software Mesquite v3.61 [92].

## 3. Results

### 3.1. Karyotype Description

**Figure 2:**
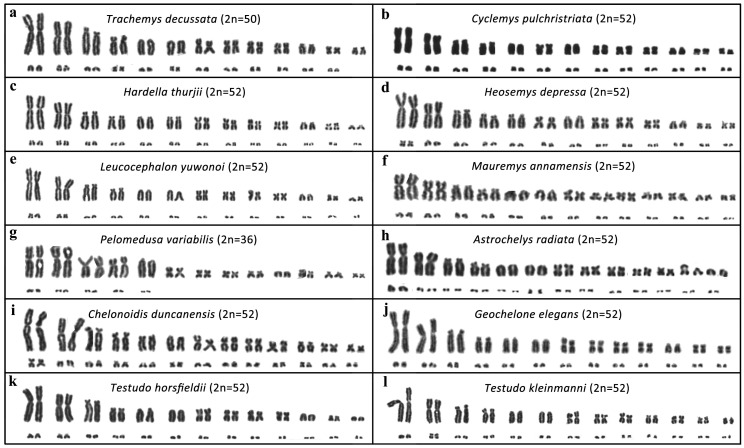
Karyograms of (**a**) *Trachemys decussata* (Emydidae), (**b**) *Cyclemys pulchristriata*, (**c**) *Hardella thurjii*, (**d**) *Heosemys depressa*, (**e**) *Leucocephalon yuwonoi*, (**f**) *Mauremys annamensis* (all Geoemydidae), (**g**) *Pelomedusa variabilis* (Pelomedusidae), (**h**) *Astrochelys radiata*, (**i**) *Chelonoidis duncanensis*, (**j**) *Geochelone elegans*, (**k**) *Testudo horsfieldii*, (**l**) *Testudo kleinmanni* (all Testudinidae).

We analyzed 30 species with karyotypes ranging from 2n = 26 in *Peltocephalus dumerilianus* to 2n = 66 in the softshell turtles *Apalone ferox* and *Lissemys punctata*. To the best of our knowledge, the karyotypes of the following 12 species are presented here for the first time (Figure 2): *Trachemys decussata* 2n = 50 (Emydidae), *Cyclemys pulchristriata* (2n = 52), *Hardella thurjii* (2n = 52), *Heosemys depressa* (2n = 52), *Leucocephalon yuwonoi* (2n = 52), *Mauremys annamensis* (2n = 52) (all Geoemydidae), *Pelomedusa variabilis* (2n = 36) (Pelomedusidae), *Astrochelys radiata* (2n = 52), *Chelonoidis duncanensis* (2n = 52), *Geochelone elegans* (2n = 52), *Testudo horsfieldii* (2n = 52) and *Testudo kleinmanni* (2n = 52) (all Testudinidae).

### 3.2. Presence of ITRs

**Figure 3:**
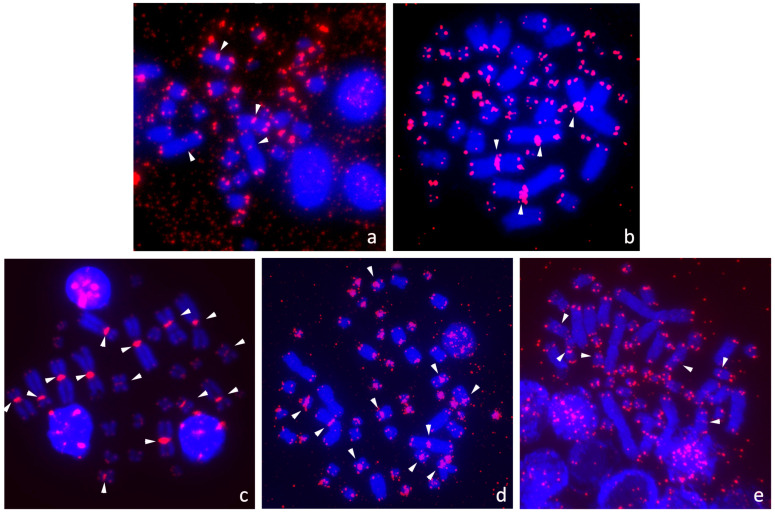
Interstitial telomeric repeats (ITRs) were detected in (**a**) *Claudius angustatus*, (**b**) *Staurotypus salvinii* (both Kinosternidae), (**c**) *Podocnemis unifilis* (Podocnemididae), (**d**) *Chelonoidis duncanensis*, (**e**) *Stigmochelys pardalis* (both Testudinidae). White arrows indicate ITRs.

**Figure 4:**
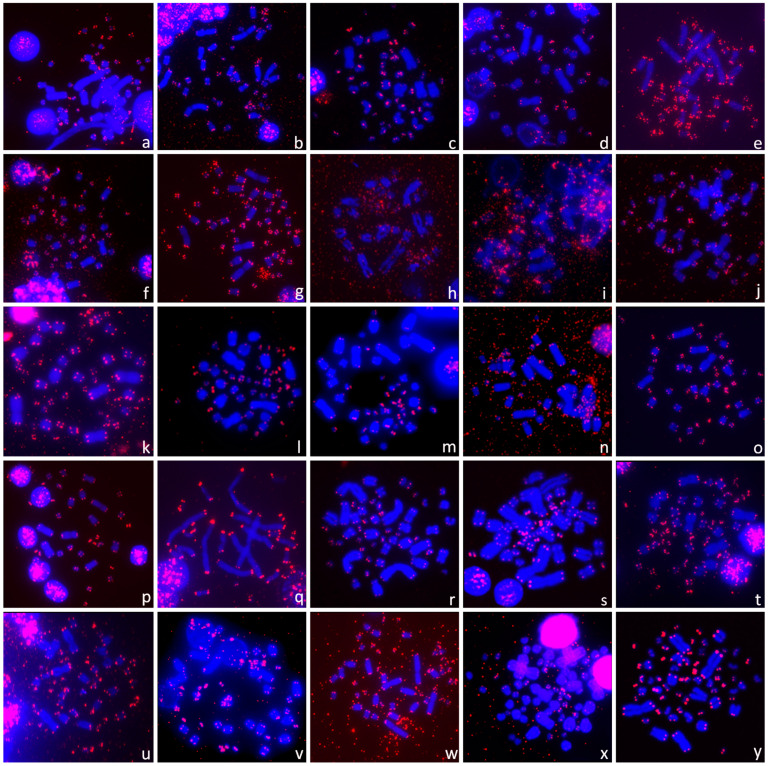
The telomeric (TTAGGG)_n_ repeats were detected only in terminal positions in (**a**) *Carettochelys insculpta* (Carettochelyidae), (**b**) *Emydura subglobosa*, (**c**) *Mesoclemmys hogei* (both Chelidae), (**d**) *Graptemys pseudogeographica*, (**e**) *Trachemys decussata* (both Emydidae), (**f**) *Cyclemys dentata*, (**g**) *Cyclemys pulchristriata*, (**h**) *Hardella thurjii*, (**i**) *Heosemys depressa*, (**j**) *Leucocephalon yuwonoi*, (**k**) *Malayemys macrocephala*, (**l**) *Mauremys annamensis*, (**m**) *Mauremys japonica*, (**n**) *Mauremys rivulata*, (**o**) *Siebenrockiella crassicollis* (all Geoemydidae), (**p**) *Pelomedusa variabilis* (Pelomedusidae), (**q**) *Peltocephalus dumerilianus* (Podocnemididae), (**r**) *Astrochelys radiata*, (**s**) *Chelonoidis carbonarius*, (**t**) *Geochelone elegans*, (**u**) *Gopherus berlandieri*, (**v**) *Testudo horsfieldii*, (**w**) *Testudo kleinmanni* (all Testudinidae), (**x**) *Apalone ferox*, (**y**) *Lissemys punctata* (both Trionychidae).

We analyzed the topology of TTAGGG repeats across a wide range of species, covering nine out of the 14 extant families of turtles. All examined species possessed multiple TTAGGG repeats in the expected terminal telomeric positions (Figure 3 and Figure 4). The telomeric signals looked brighter on microchromosomes than on macrochromosomes in almost all species.

We detected ITRs in only five out of the 30 examined species and they were located exclusively in the centromeric positions (Figure 3). ITRs were detected at the centromeres of chromosomes 1 and 2 in *Claudius angustatus* and *Staurotypus salvinii* (Figure 3a,b). In *Podocnemis unifilis*, ITRs accumulated at the centromeres of chromosomes from the seven largest pairs (Figure 3c), confirming reports from previous studies [76,78]. In *Chelonoidis duncanensis*, ITRs were detected in the centromeric regions of chromosome pairs 1, 4, 8 and 9 (Figure 3d). In *Stigmochelys pardalis*, ITRs were present in the centromeres of chromosome pairs 6, 8 and 9 (Figure 3e).

### 3.3. Distribution of ITRs across the Turtle Phylogeny

Pooling together our results with data from previous studies, ITRs were identified in 10 out of 55 examined turtle species. The phylogenetic pattern of the topology of TTAGGG motifs across turtle genomes suggested that ITRs evolved independently at least seven times (Figure 1).

## 4. Discussion

All examined turtle species possessed multiple telomeric repeats in terminal positions. We detected ITRs in five species, increasing the total number of known turtle species with ITRs to 10. ITRs seem to be restricted to the centromeres, except for the Y chromosome of *Elseya novaeguineae*, where telomeric-like sequences were amplified in the intermediate position of the p-arm (Figure 1). Interestingly, as in birds [93,94,95,96,97] and squamate reptiles [28,51,98], in almost all examined turtle species the telomeric signals look brighter on microchromosomes than on macrochromosomes. This stronger signal brightness has been previously reported in other vertebrate lineages, including birds [39] and squamates [28,48,98], and it can be caused by a higher number of telomeric repeats on microchromosomes [94]. The species analyzed in the present study cover a large part of turtle phylogenetic diversity from both suborders (Pleurodira and Cryptodira) and nearly the whole variability in the diploid chromosome numbers known in turtles. Despite this variability, only 18.2% of the studied turtle species have ITRs detectable with molecular cytogenetic methods (Figure 1). Phylogenetic distribution suggests that ITRs evolved independently within turtles at least seven times.

Kinosternidae are reported to have undergone minimal chromosomal changes [99,100], where the main difference between the subfamilies Staurotypinae (2n = 54) and Kinosterninae (2n = 56) is found in a pair of microchromosomes. Centromeric ITRs have been described for the two species of the genus *Staurotypus* and their sister taxon *Claudius angustatus* (Figure 3a,b and Figure 1). The only other species from the family Kinosternidae analyzed up to now, *Sternotherus odoratus* (2n = 56), does not have any ITRs (Figure 1). It seems that the centromeric ITRs are an apomorphy of the subfamily Staurotypinae and cannot be directly connected to chromosome rearrangements leading to formation of the derived karyotype of the family Kinosternidae or its subfamily Staurotypinae.

A burst of ITRs emerged in *Chelydra serpentina* (Chelydridae), where they are present in seven chromosome pairs, although this species possesses with 2n = 52 the putatively ancestral diploid chromosome number for turtles [76] (Figure 1).

The presence of ITRs in centromeres in *Podocnemis unifilis* (Figure 3c) [76,77,78] and *P. expansa* [78] was suggested to be a remnant of ancestral fusions leading to the lower diploid chromosome number in the family Podocnemididae [76,77,78]. Nevertheless, the situation is more complicated. In *P. expansa*, Noronha et al. [77] detected telomeric sequences only in the terminal positions, while Cavalcante et al. [78] reported ITRs with a similar pattern as in the closely related *P. unifilis*. ITRs can contain a different number of telomeric-like repeats, which might be reflected in the intensity of the probe signal, and there might be interindividual variability in the number of repeats. Nevertheless, such a strong difference within the same species is surprising. None of the two studies reported a genetic identification of the examined specimens (e.g., mtDNA sequence per specimen), leaving open the possibility that perhaps due to taxonomic misidentification, different taxa were examined in each study. Regardless of this questionable report, the lack of ITRs in *Peltocephalus dumerilianus*, another member of family Podocnemididae, suggests that the presence of ITRs is not directly connected to fusion events. Supporting this view, ITRs have not been found in *Pelomedusa variabilis* (Figure 1), as well as in *Pelomedusa subrufa* [76] (Figure 1), even though it is possible that a different taxon was studied because formerly all ten currently recognized species [87,101] were lumped together as *Pelomedusa subrufa*. In any case, the genus *Pelomedusa* belongs to the family Pelomedusidae, the sister taxon of Podocnemididae. Pelomedusids possess a similar or even lower diploid number compared to podocnemidids, and the reduction in chromosome number can thus be a synapomorphy of Pelomedusidae and Podocnemididae. If in the common ancestor of these two families the fusions responsible for the reduction of the chromosome number would have led to ITRs in the centromeric region, the ITRs had to be retained for a long evolutionary time in some species, while they were lost in others.

A connection between ITRs and chromosomal fusions is also not supported for the family Testudinidae. Previous studies did not detect ITRs in *Chelonoidis carbonarius* and *C. chilensis* [72,75]. In *Chelonoidis duncanensis*, we detected centromeric ITRs in four chromosome pairs (Figure 3d), although all three species of the genus share the same chromosome number 2n = 52. We also detected ITRs in centromeres of three chromosome pairs in *Stigmochelys pardalis*, another species sharing the same chromosome number (2n = 52) typical for the family Testudinidae (Figure 3e). The phylogenetic position and the different topology of the accumulation of telomeric-like repeats suggest that ITRs evolved within testudinidids independently in *C. duncanensis* and *S. pardalis* (Figure 1). 

Most turtles have environmental sex determination (ESD) [76]. As far as we know, genotypic sex determination (GSD), and hence sex chromosomes, evolved independently at least five times within turtles: ZZ/ZW sex chromosomes in the common ancestor of softshell turtles (family Trionychidae [102]), XX/XY sex chromosomes in the genus *Glyptemys* (family Emydidae [103], in the genus *Staurotypus* (family Kinosternidae [104,105]), in *Siebenrockiella crassicollis* (Geoemydidae [106]) and in chelid turtles (reviewed in [81]).

Non-recombining parts of the unpaired W and Y chromosomes tend to degenerate, i.e., to lose functional genes [107,108,109], and to accumulate repeats such as microsatellites [49,53,56,62,110,111], rDNA-derived repeats [50,73,81,112] and telomeric-like repeats [27,28,61,113,114,115]. In turtles, the Y chromosome is not enriched in telomeric-like sequences in *Glyptemys insculpta* [76], *Staurotypus salvinii* and *S. triporcatus* [76]. Similarly, a degenerated W chromosome in trionychid turtles is full of rDNA-derived repeats, but no accumulation of telomeric-like repeats was detected in *Apalone ferox*, *Lissemys punctata* (Figure 4x,y), *Apalone spinifera* or *Pelodiscus sinensis* [76]. Within chelids, ITRs are present on the Y chromosomes in *Elseya novaeguineae* [81] but not on the putatively homologous Y chromosomes of the genera *Chelodina* and *Emydura* [76,81]. The comparison of repeat content on unpaired sex chromosomes across turtles suggests that it can be very variable and can reflect historical contingency rather than a functional aspect of a given repeat. 

ITRs occur only rarely in turtles, especially in comparison to squamates [28]. Wherever present in turtles, ITRs are restricted to centromeric regions and to the non-recombining parts of sex chromosomes (Figure 1). The distribution of ITRs agrees with the observation that turtle karyotypes are very conserved [85] and that centromeres and non-recombining parts of sex chromosomes are the most dynamic parts of genomes [116,117]. Centromeric ITRs do not seem to be directly related to interchromosomal rearrangements in turtles. If ITRs were formed during fusions, they had to be subsequently lost in several cases. Such a scenario was supported, for instance, by Nanda et al. [39] for birds, possibly due to selection for smaller genome size counteracting the accumulation of repetitions and resulting in the disappearance of ITRs. That the presence of centromeric ITRs is not related to rearrangements is further supported by the fact that in species possessing ITRs, the number of chromosomes with ITRs does not agree with the estimated number of interchromosomal rearrangements for the respective karyotype. However, our conclusions on the homology of chromosomes with ITRs are based on the comparison of chromosome morphology, and should be further tested using more informative approaches, such as comparative BAC-FISH or chromosome painting.

Interestingly, although ITRs are generally rare in turtles, whenever they occur, they tend to be present in centromeres in more than one chromosome pair. ITRs are present in two chromosome pairs in the kinosternids *Claudius angustatus* and *Staurotypus salvinii*, in three chromosome pairs in *Stigmochelys pardalis*, in four chromosome pairs in *Chelonoidis duncanensis*, and even in seven chromosome pairs in *Podocnemis unifilis* and *Chelydra serpentina* (Figure 1; [76]). This observation can be attributed to a phenomenon known as “centromere homogenization” potentially caused by the involvement of telomeric-like repeats in retrotransposons colonizing centromeres [118]. Another interesting observation is that only centromeric ITRs were detected in turtle autosomes. One reason can be that centromeric ITRs are the most common type, at least in squamates [28]. It is therefore likely that they would be overrepresented also in the few cases of ITRs found in turtles. However, it is also possible that in turtles, events leading to ITRs within chromosome arms are rarer. Intermediate ITRs have been found in squamates [28] and in birds [39], and it was speculated that they were formed due to chromosome inversions, which are common in these groups. Turtles, thus, may be less prone to intrachromosomal inversions than squamates [119] and birds [97,120]. As these inversions are important for the evolution of postzygotic reproductive isolation mechanisms, and thus speciation [120,121,122,123,124], this feature may contribute to the generally low species diversity in turtles [70] and their slow molecular evolution [125,126,127]. The low rate of inversions in the karyotype evolution of turtles could explain why they exhibit a high incidence of genomic introgression and why they extensively hybridize (and are often able to produce fertile offspring), even after many million years of divergence, as documented in chelid, cheloniid and geoemydid turtles [81,91,128,129,130,131,132,133,134,135,136]. 

In turtles, the TTAGGG motif is restricted only to the centromeric regions. The lack of a strong phylogenetic signal in the distribution of ITRs across all studied species (Figure 1) indicates that the telomeric-like sequences in the centromeres of turtles are not connected to chromosomal rearrangements. Nevertheless, the identification of ITRs is a more complicated process. Genome sequencing projects revealed that short arrays of ITRs, below the detection efficiency of in situ hybridization methods, are common in vertebrate genomes [19,20,21,22,23,24,25,26]. Such s-ITRs are expected to exist in turtle genomes as well. Furthermore, long ITRs, e.g., derived from chromosome fusions, are non-essential, non-coding regions, and like other microsatellite motifs, they can degenerate over time by reduction of copies and/or by accumulation of mutations, which will complicate their detection by in situ hybridization or bioinformatic methods. Previous studies in rodents [27] and birds [68,69] documented that the TTAGGG motif can be part of a longer satellite sequence occurring in centromeres and other heterochromatic regions. Notably, a telomeric-like TCATGGG motif forming long tandem repeats has been identified in the pericentromeric regions of *Drosophila hydei*, and this motif does not seem to have a telomere-related function [137], suggesting that this could also be the case in turtles with ITRs.

In conclusion, the centromere organization and sequence composition in turtles are currently not well known. This calls for further research to understand whether or not the TTAGGG motif in the centromeres of turtles has a non-telomeric origin, e.g., using long-read throughput sequencing of the centromeric regions of phylogenetically informative species.

## Figures and Tables

**Table 1:** Summary of species analyzed in this study.

Suborder	Family	Species	Sex		
			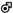	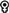	Unknown
Cryptodira	Carettochelyidae	*Carettochelys insculpta*	-	-	1
	Emydidae	*Graptemys pseudogeographica*		1	
		*Trachemys decussata*	1	-	-
	Geoemydidae	*Cyclemys dentata*	-	-	1
		*Cyclemys pulchristriata*	2	-	-
		*Hardella thurjii*		1	
		*Heosemys depressa*	-	1	-
		*Leucocephalon yuwonoi*	1	1	-
		*Malayemys macrocephala*	1	-	-
		*Mauremys annamensis*	2	2	-
		*Mauremys japonica*	-	-	4
		*Mauremys rivulata*	-	1	-
		*Siebenrockiella crassicollis*	-	1	-
	Kinosternidae	*Claudius angustatus*	1	1	-
		*Staurotypus salvinii*	1	-	-
	Testudinidae	*Astrochelys radiata*	-	-	1
		*Chelonoidis carbonarius*	-	-	1
		*Chelonoidis duncanensis*	1	-	-
		*Geochelone elegans*	1	1	-
		*Gopherus berlandieri*	1	-	-
		*Stigmochelys pardalis*	1	-	-
		*Testudo horsfieldii*	-	1	-
		*Testudo kleinmanni*	1	-	-
	Trionychidae	*Apalone ferox*	-	1	1
		*Lissemys punctata*	-	1	-
Pleurodira	Chelidae	*Emydura subglobosa*	-	1	-
		*Mesoclemmys hogei*	1	-	-
	Pelomedusidae	*Pelomedusa variabilis*	1	1	-
	Podocnemididae	*Peltocephalus dumerilianus*	1	-	-
		*Podocnemis unifilis*	1	-	-
